# Money, morality, and nationalism: the contested sponsorship of the Falsterbo horse show

**DOI:** 10.3389/fspor.2026.1741781

**Published:** 2026-04-13

**Authors:** Jesper Karlsson

**Affiliations:** Research in Education & Movement Culture, Swedish School of Sport and Health Sciences, Stockholm, Sweden

**Keywords:** affective economies, national identity, postcolonial imaginaries, sponsorship, sport management, sportwashing

## Abstract

In early 2025, the Falsterbo Horse Show entered a title sponsorship agreement with Al Shira'aa Stables in the United Arab Emirates, prompting extensive Swedish media coverage and strong public reactions. This controversy offers an opportunity to examine how sport sponsorship becomes a site for moral and geopolitical boundary-making. Drawing on a Foucauldian understanding of discourse as a meaning-making practice, the study analyses 210 Swedish newspaper articles published between February and April 2025 to explore how power/knowledge operates through language and how sponsorship is discursively framed. The analysis identifies three interconnected discourses: the sold-out soul and sportwashing, dirty money and postcolonial morality, and pure money and Swedish nostalgia, that frame the sponsorship as morally and geopolitically contentious. Across these discourses, processes of fetishization and reduction imbue sponsorship funds with moral significance based on their origin. Media portrayals construct money from the UAE as symbolically contaminated, linked to gender inequality, LGBTQ+ rights, human rights violations, and animal welfare, while simultaneously idealizing Swedish sponsorship through nostalgic narratives of national integrity and moral coherence. These judgements draw on broader affective and historical formations, including white melancholia, which position Sweden as a nation losing an imagined ethical distinctiveness. Rather than evaluating the sponsorship itself, the article shows how sponsorship functions as an affective practice shaped by capitalism's identity-producing machinery, delineating which sponsors become imaginable or legitimate. By situating media reactions within wider cultural and geopolitical imaginaries, the study contributes to sport management and sponsorship research by showing how sponsorship is implicated in discourses of morality, identity, and belonging.

## Introduction

In early 2025, one of Sweden's oldest and most prominent equestrian events, the Falsterbo Horse Show, announced a sponsorship agreement with Al Shira'aa Stables. The deal included a renaming of the event to the Al Shira’aa Falsterbo Horse Show. Media coverage following the announcement largely focused on the fact that Al Shira’aa Stables is a company based in the United Arab Emirates (UAE) with clear ties to the country's ruling royal family, connections that were perceived to conflict with so-called “values commonly framed as ‘Swedish’.” The criticism became so extensive that the Falsterbo Horse Show chose to terminate the partnership just 1 month after it was made public.

The reactions to the sponsorship offer insight into how sponsorships can be entangled with implicit moral dilemmas for the sponsored organization. This perspective is largely absent in previous sponsorship research, which has primarily focused on the relationship between sponsors and the sponsored organization, or between the sponsored organization and fans or consumers, and whether a sponsor is deemed appropriate or inappropriate from an economic standpoint (1–6). While this type of research is relevant for understanding sponsorship relations, it lacks social science perspectives that could offer alternative perspectives on the phenomenon. Such perspectives can broaden the analytical lens beyond economic interpretations and enable analyses that consider how historical and cultural contexts shape sponsorships. Without such understanding, research risks oversimplifying sponsorship relations and overlooking how capital is deeply embedded in historical, cultural, social, and affective discourses (7–9). By expanding the perspective and applying a critical social science approach to sponsorship, research can trace how market relations, of which sponsorship is a part, are never neutral (8–10, 60).

In this article, the aim is to contribute to a critical perspective on sponsorship relations by analyzing the Swedish news media coverage of the sponsorship agreement between Falsterbo Horse Show and Al Shira'aa stables. Drawing on a postcolonial theoretical perspective, complemented by critical theories of capitalism, the guiding research question is: How is the sponsorship portrayed in Swedish news media coverage, and how do these portrayals reproduce postcolonial imaginaries of Sweden and the United Arab Emirates?

## Previous research

### Sport sponsorship—a relational practice

In today's sports landscape, it is rare to find teams, competitions, arenas, or events that are not sponsored by corporate entities. Research has shown that companies perceive numerous strategic advantages in sponsoring sports, such as the opportunity to engage more directly with potential customers (11), to differentiate their brand from other market actors (12), to build corporate image and identity (11), and to increase brand awareness and loyalty (13, 14). Sponsorship thus emerges as a multifaceted strategy for branding and marketing, both locally and globally.

At the same time, sponsored organizations have their own objectives for entering sponsorship agreements. In sports, these often include raising awareness of the sport, enhancing their image, selling more tickets and merchandise, and reaching a broader audience (15). These effects can, in turn, make the club more attractive to future sponsors and media partners (16). Sponsorship, therefore, not only enables financial survival for sports clubs and events (17, 18) but can also contribute to positive image transfers and enhance audience experience (5, 19–21).

Research also emphasizes that choosing the right partner is crucial, not only for the sponsor but also for the sponsored organization. Sponsorship can generate strong positive effects. Thomas et al. (22), for example, highlight Red Bull's sponsorship of Felix Baumgartner's stratospheric parachute jump, which was viewed by over nine million people, an impact that likely would not have been possible without the sponsor's involvement. On the other hand, several studies show that a sponsor's behavior, or even mere presence, can provoke negative reactions if the company is not appreciated by fans or consumers (3, 6, 23). Sponsorship thus appears as a relational practice, in which sponsors, the sponsored organization, and fans or consumers mutually influence one another (24, 25). This is, for instance, shown in previous research that name changes of arenas, often a result of sponsorship, can provoke negative reactions among supporters. These reactions are frequently expressed as feelings of loss. Supporters may feel that identity, history, and local rootedness have been sold out, and some even express a willingness to pay higher ticket prices to preserve what it once was (1, 4).

Beyond concerns connected to tradition, fans also react to sponsorships on moral grounds. One reason fans react positively or negatively to sponsorships is their perception of the sponsor's moral appropriateness in sponsoring a sports team or sporting event (26). Fans and consumers may feel that companies operating in controversial markets, such as those involved in unhealthy food, alcoholic beverages, tobacco, and polluting industries, use sponsorships to leverage the positive image that sports generate, both through the sport itself and through the team, to benefit their own brand (27, 28).

Another area closely related to this is what some researchers have termed soft power sports sponsorship (e.g., 29). Chadwick et al. (29, p. 200) define it as “a contractual relationship between a fully or partly state-owned entity and a property aimed at promoting the attractiveness of the former's country, culture and/or policies, and with the intention of altering the attitudes and behaviors of key target audiences (which may include overseas politicians, decision-makers, as well as customers)”. However, their primary interest lies in analyzing this phenomenon through social network analysis, focusing on how states and companies are structurally linked rather than on how such sponsorships are perceived. Reiche (30) examines, though, how fans of the football clubs FC Barcelona, Bayern Munich, and Paris Saint-Germain perceive sponsorship deals with Qatar Airways and other Qatar-related companies owned by the ruling family of Qatar. Supporters of both FC Barcelona and Bayern Munich protested these sponsorships, citing Qatar's lack of respect for human rights, whereas the sponsorship was not seen as particularly controversial among Paris Saint-Germain fans. Reiche suggests that this may be because, in the case of Paris Saint-Germain, the purpose of the sponsorship was to contribute to future success, while in the cases of Barcelona and Bayern Munich, the goal was rather to maintain existing success. In other words, the perceived purpose of a sponsorship deal appears to influence whether it is considered morally questionable.

What is missing from the research, however, is an understanding of how reactions to sponsorship deals are entangled in capitalism's identity-producing machinery (7, 8) and how this machinery shapes ways of feeling, thinking, and acting. In the next section, I deepen this discussion by examining capitalism as a system that generates differences and boundaries through ongoing processes of identity production.

## Theoretical points of departure

In sport management research, Chen (31) argues that a ghost is haunting the field. He describes this ghost as capitalism, an ever-present force that is rarely made explicit in sport management research. Chen emphasizes the importance of making capitalism's structure visible. I share these perspectives but seek to further develop and deepen the understanding, both to complicate the picture and to clarify how I conceptualize capitalism in relation to sponsorship in general, and specifically in the case of the sponsorship agreement between the Falsterbo Horse Show and Al Shira'aa Stables.

I view capitalism as a complex machine, a network of relations, bodies, places, and affects that shape how we feel, act, and perceive ourselves. Capitalism is not merely an economic system; it is also a cultural system that produces differences and boundaries, through which identities are formed (7–9, 32–34). In other words, the capitalist system is an identity-producing machine that creates divisions among people, nations, and regions, as well as within the world of sport. As Hall (60, p. 185) puts it: “Our ideas of ‘East’ and ‘West’ have never been free of myth and fantasy, and even to this day they are not primarily ideas about place and geography.” The differences and boundaries produced by the capitalist system are therefore not only about geographical distance (9, 60). They also concern ideas about those on the other side of the boundary, and those within it. This has long been emphasized by postcolonial theorists (10, 35–37).

A central aspect of capitalism's production of difference is that the West, in addition to exploiting the resources of other regions, has also produced narratives that legitimize this exploitation. These narratives have often relied on a dichotomy between West and East, where the West is portrayed as civilized, rational, modern, and egalitarian, while the East is portrayed as traditional, irrational, exotic, patriarchal, undemocratic, and threatening (10, 36–38, 60). Since September 11, 2001, these narratives have intensified. Muslims from the Middle East have increasingly been portrayed as a potential threat to Western values, not only in terms of security, but also culturally and emotionally (39–42).

Another important dimension of this boundary-making process is the cultural imaginaries of purity and dirtiness that run throughout postcolonial theory (38, 42, 43). Notions of “dirt” and “purity” do not refer to material cleanliness but to symbolic and moral classifications that mark certain bodies, cultures, and practices as contaminated or threatening. Following Saunders-Hastings (44), I use the concepts of fetishization and reduction not to assess the moral legitimacy of financial exchanges, but to analyze how media discourse assigns fixed moral qualities to money and simplifies complex political and economic relations.

Introducing this lens makes it possible to understand how sponsorship money becomes a site where postcolonial hierarchies, national identity, and capitalist economic systems are entangled. In this study, I employ the concepts of dirty money and pure money analytically to examine how these moralized differences operate in the Swedish media discourse.

## Methodology

Building on the theoretical framework outlined above, this study employs a Foucauldian discourse-analytical approach guided by his understandings of language, power, and knowledge. Discourses are understood here as systems of portrayals, patterns of recurring statements, concepts, and formulations that together construct a particular understanding of a phenomenon (45). Discourses are not neutral; they produce knowledge that shapes how we perceive the world, ourselves, and others. They regulate what can be said, thought, and done within a given field, and are therefore always linked to power (45, 60). Discourses thus have real consequences; they affect not only how we speak about the world but also how we act within it (60). This perspective enables the analysis of how Swedish news media produce particular imaginaries of the sponsorship agreement between the Falsterbo Horse Show and Al Shira'a Stables.

### Data production

Another methodological implication of the above concerns the understanding of data. From this perspective, data should not be viewed as something that exists “out there,” waiting for me as a researcher to collect, organize, or determine its true meaning. Such an assumption rests on onto-epistemological premises that differ from those shaping this article. Instead, data are always already entangled with the researcher (46).

In this sense, the dataset used in this article did not originate from a detached decision to “collect” material, but rather from a series of encounters (a force from the outside that forced me to think) (47). The knowledge produced is therefore partial and situated (48). The Falsterbo Horse Show's sponsorship agreement with Al Shira'aa Stables first became visible to me through television news, where it was reported that the sponsorship had received criticism on social media. This made me curious about how the criticism was expressed, and I observed that it drew heavily on postcolonial imaginaries, with clear parallels to narratives reproduced by Western media ahead of and during the FIFA World Cup in Qatar (49). Due to ethical considerations regarding the use of social media posts from private individuals, I chose instead to examine how the issue was covered in Sweden's major daily newspapers. From reading the articles, I noticed that the sponsorship deal carried a range of ideas about Al Shira'aa Stables and the UAE, which led me to read all relevant articles retrieved from the Swedish news database *Mediaarkivet*.

The articles were those returned by the query “Falsterbo Horse Show Al Shira’aa”. The articles were published between February 24, 2025, the day the sponsorship agreement was announced, and April 30, 2025, a point in time after the agreement had been terminated. This timeframe was selected to capture both the immediate reactions to the announcement and the subsequent development of media coverage. It quickly became evident, however, that the most intense reporting occurred around the time of the announcement. By late April, when the agreement had been terminated for about a month, media coverage about the sponsorship deal had significantly declined.

Since *Mediaarkivet* aggregates material from a wide range of Swedish news outlets, the search produced substantial data material. In total, 210 articles are included, of which 75 are so-called rewrites, articles republished across multiple newspapers. Although these are duplicates, they indicate the extent to which coverage was disseminated nationwide.

This study analyzes only verbal newspaper text, that is, the written language of the news articles. Other forms of expression, such as images, layout, or typography, have not been included in the analysis.

### Analytical approach and process

The analysis focuses on how power operates through media portrayals, that is, how language regulates understandings of the world and shapes subjectivities (45, 60). As Foucault put it (50, p. 182), “For power relations, discourse is not merely a surface onto which signs can be attached, it is an operator”. In the results, this is emphasized by showing how discourses operate as active forces in the construction of meaning, thereby shaping perceptions of “the world”, “of others”, and “of us”.

Describing a Foucauldian discourse analysis as a strictly linear or schematic procedure is methodologically difficult. Rather than following a predetermined sequence of coding steps, the analysis is guided by what Bryman (51) calls an analytic mentality, a way of reading in which theory and data continually inform one another. This means that I had already adopted my theoretical and methodological points of departure during the initial reading of the articles. Following Jackson and Mazzei (52) on thinking data with theory, theoretical perspectives did not function as a coding template. Instead, this approach made it possible to attend not only to explicit statements but also to discursive silences and underlying assumptions.

Discursive formations were not identified based on recurrence but on their productive capacity to structure meaning. Analytical significance was therefore tied to the work a formation performed in organizing moral boundaries within the material, rather than to the number of fragments in which it appeared. Alternative interpretations were considered throughout the reading process, and fragments were evaluated in relation to broader patterns of meaning rather than by quantitative weight.

Another way to describe this analytical process is that, as I read the articles, I extracted the text fragments that “glowed”, as MacLure (53) puts it. These text fragments were selected for their relevance to the research questions and resonance with the theoretical and methodological framework. At the same time, discourse analysis, like all analysis, requires methodological transparency. The following section, therefore, attempts to outline the process by which the analysis was conducted more concretely.

The analysis proceeded in several stages. First, all articles were read in full to gain an overview of the material. During this initial phase, I noted recurring themes, formulations, and meaning-bearing patterns in the texts. These excerpts formed the discourse fragments: small analytical units that capture central ways of speaking about, valuing, and understanding the sponsorship agreement and its associated actors. These discourse fragments included, but were not limited to, excerpts describing the sponsorship deal between the Falsterbo Horse Show and Al Shira'aa Stables, excerpts describing Al Shira'aa Stables and its relationship with the ruling elite in the United Arab Emirates, and excerpts describing the differences between the United Arab Emirates and Sweden.

In the next stage, a close reading of these discourse fragments was conducted. In this stage, I focused on how they interacted to form discursive formations. This process also involved tracing how descriptions, such as references to “selling one's soul” or “sportwashing”, circulated across different newspapers and reporting genres, and how these fragments contributed to the construction of moral discourses about the origins of sponsorship money.

Based on this in-depth analysis, I developed three overarching analytical questions: How are discourses of the sponsorship reproduced in the media? How are discourses about the UAE reproduced? And how are discourses about Sweden reproduced? These questions provided the structure for constructing the fragments into three broader discursive formations.

The first formation concerned portrayals of moral betrayal and sportwashing, captured in the sold-out soul and sportwashing discursive formation. The second centered on the moralization of financial flows and the construction of cultural difference between the UAE and Sweden, which I refer to as the dirty money and postcolonial moralism discursive formation. The third focused on the idealization of Swedish sponsors and the nostalgia surrounding an imagined tradition of national integrity, conceptualized as the pure money and Swedish nostalgia discursive formation.

In the results section, I refer to these formations simply as discourses. They reflect not only recurring linguistic patterns but also the assumptions, values, and boundaries operating within the media coverage in their portrayal of the sponsorship deal. Through this combined interpretive and procedural approach, the analysis remained situated in empirical material while simultaneously attending to the theoretical and methodological approaches that shaped the interpretation. Consequently, the results are presented in a narrative style, with empirical excerpts, interpretive commentary, and theoretical insights interwoven. Rather than treating theory as a separate layer of “explanation,” this analytical approach allows theoretical insights to appear through the data (46), showing how the discourses operate as an active force in the Swedish media's portrayal of the Falsterbo Horse Show sponsorship.

### The sold-out soul and sportwashing

In the following section, I present the three discourses that emerged from the analysis and show how they operate in the media coverage of the sponsorship agreement and the actors involved.

One discourse that operates in the portrayal of the sponsorship agreement between the Falsterbo Horse Show and Al Shira'aa Stables is the notion that Falsterbo Horse Show has sold its soul. This discourse is framed by the renaming of the event to Al Shira'aa Falsterbo Horse Show. For example:

Falsterbo Horse Show changes its name to Al Shira'aa Falsterbo Horse Show following a new title sponsorship agreement with a sponsor from the United Arab Emirates. After the announcement, the loyal equestrian audience erupts in protest, claiming the event has sold its soul. (Sydsvenskan, February 26, 2025)

The discourse of the sold-out soul operates through strong emotions that, at first glance, could be interpreted as a sense of loss of tradition associated with the event. However, this discourse does not operate solely through concerns about the sale or the renaming of the event itself; it also operates as a moral boundary-making practice, focusing on the sponsor with whom Falsterbo Horse Show has entered into an agreement. In this context, Al Shira'aa Stables is portrayed as a company that leverages equestrian sport to enhance the UAE's reputation. In the media coverage, this is made explicit, for example, in the following excerpt:

The name change and choice of sponsor have already sparked reactions. The event is accused of having sold its soul, and part of the criticism concerns Falsterbo Horse Show's contribution to sportwashing. (Aftonbladet, February 26, 2025)

As shown in the excerpt, the discourse of the *sold-out soul* is entangled with notions of sportwashing, linking the sponsorship deal to moral corruption and manipulation, thereby reinforcing a West/East dichotomy and reproducing narratives criticized by postcolonial scholars (10, 36–38).

In general, Swedish media portrayals of the sponsorship agreement are similar, though there are variations in how explicit they are in their critique, as seen in the following excerpt:

Falsterbo Horse Show has changed its name to Al Shira'aa Falsterbo Horse Show, after securing a new main sponsor with close ties to the dictatorship in the United Arab Emirates. One of Sweden's most prestigious equestrian competitions has become part of a global sportwashing effort, where money from one of the world's most oppressive regimes is used to buy respect and prestige in the West. (Sydsvenskan, March 1, 2025)

In other words, it appears that the sold-out soul discourse operates through postcolonial imaginaries and is entangled with notions that embed the sponsorship in broader geopolitical dynamics.

In the next section, I trace how these geopolitical dynamics are entangled with the discourse of money, portrayed as tainted or contaminated. I show how this can be understood as part of capitalism's identity-producing machinery, which operates as a system that reproduces differences and boundaries (7, 9, 32), and how these boundaries are further reinforced through postcolonial imaginaries.

### Dirty money and postcolonial morality

The previous section showed how the sold-out soul discourse operates through postcolonial imaginaries. A related but distinct imaginary emerges in the discourse of dirty money. Here, the UAE, and by extension Al Shira'aa Stables, is portrayed as fundamentally different from Sweden, with its proximity to the ruling royal family framed as morally compromising. Through this logic, the sponsor's money is discursively marked as “dirty.”

In the media portrayal of the Falsterbo Horse Show and Al Shira'aa Stables sponsorship agreement, this discourse operates by depicting certain sources of money as more or less acceptable than others. In an article from southern Sweden, referencing another regional newspaper, this is described as follows:

Sydsvenskan has investigated the origin of the horse show's new sponsorship money: An unimaginably wealthy dictator's family, maintaining a strict dictatorship with sharia law, where homosexuality can be punished by death. They oppress migrant workers and wage a bloody war in Yemen. (Helsingborgs Dagblad, March 1, 2025)

This excerpt shows how the sponsorship money is portrayed as contaminated due to Al Shira'aa Stables' connection to the ruling royal family in a country described as violating human rights, including the persecution of homosexual individuals and, more broadly, sexual and gender minorities, oppressing migrant workers, and engaging in warfare. This reflects a process of fetishization and reduction, in which the money's origin is imbued with a fixed moral essence, and the donor is collapsed into a simplified moral category. As Saunders-Hastings (44) notes, such interpretations overlook the relational nature of wealth. Here, the political and social complexity of the UAE, and of the sponsorship system itself, is reduced to “an unimaginably wealthy dictator's family,” reproducing postcolonial imaginaries that associate certain regions and cultural Others with moral contamination. This reduction flattens not only the geopolitical context but also the structural dynamics of the sponsorship system, obscuring the broader capitalist conditions that shape all sponsorship transactions.

The discourse of dirty money is also reinforced by politicians who comment on the sponsorship agreement. In a statement from a representative of a conservative youth organization, the sponsorship deal is described as morally wrong because, according to him, it originates from what he calls Islamists:

I think it's morally wrong that they choose to sell themselves in this way to Islamists. With its name, they become a symbol of the oppression of minorities in the United Arab Emirates and the sharia laws they enforce. (GT, February 26, 2025)

The discourse of dirty money operates here by the notion that the sponsorship agreement will make Falsterbo Horse Show a symbol of the oppression attributed to the UAE. There is also a discursive shift in the statement from an agreement between a Swedish event and a stable to an agreement between a nation of “Islamists” and the Falsterbo Horse Show. This exemplifies, in another way, how the discourse of dirty money takes on symbolic meaning and operates through postcolonial imaginaries where regions, regimes, or cultural Others are associated with dirty money. This also portrays the Middle East and Muslims as countries that pose a threat to Western values (39–41).

This entanglement of fetishization and reduction of money and postcolonial imaginaries within the discourse of dirty money also operates in more explicit ways. In an article from a major Western Swedish newspaper, it is described:

In a column in the magazine Ridsport, she attacks Falsterbo, accusing it of selling its soul to the devil. It's at the expense of the young equestrian girls. We’ve raised them to be free, strong, and independent. With this, they're exposed to a completely different view of women. Which also comes in as a savior that allows their favorite event to continue. It's “womanwashing.” She [the owner of Al Shira'aa Stables] is part of an old culture that is far from our own. I’m definitely not racist—it's just that this is a clash in my eyes. (GT, March 15, 2025)

This excerpt shows how the discourse of dirty money operates through the fetishization and reduction of money and the postcolonial imaginaries, reproducing a notion of difference between Sweden and the UAE. The writer emphasizes not being racist but rather being concerned with cultural differences between Sweden and the UAE. However, by portraying Al Shira'aa Stables' female representation as part of what is perceived as “womanwashing” and as a relic of an outdated culture, far removed from the Swedish, the UAE is depicted as traditional, patriarchal, and manipulative, while Sweden is portrayed as modern, egalitarian, and trustworthy.

In another article, the discourse of dirty money operates in a similar way. Here, concerns are voiced about the treatment of animals and women in the UAE:

There's been a lot of writing about how horses and animals are treated, and it's also an emirate with a view of women that is so far from our own. And that's been the case for many years. It's not something to hide, and we have to consider what kind of role models we're creating for our youth. (Kristianstadsbladet, March 1, 2025)

This excerpt shows how the dirty money discourse is entangled with gendered difference and animal welfare concerns, and how these are mediated by the fetishization and reduction of money as well as postcolonial imaginaries.

In this section, I have shown how the discourse of dirty money operates and how it is deeply entangled with capitalism's identity-producing machinery (7–9). The discourse draws on postcolonial imaginaries that portray the UAE as fundamentally different from Sweden.

### Pure money and Swedish nostalgia

Building on the previous section, this part traces how the imaginaries of fetishization and reduction also operate within the discourse of pure money. Unlike the dirty money discourse, which marks UAE wealth as morally contaminated, the pure money discourse idealizes “Swedish money” and mobilizes nostalgic imaginaries of a morally coherent national past. This is expressed in a southern Swedish morning newspaper as follows:

Horses are expensive. Very. Riders at higher levels, as well as organizers, depend on generous horse owners and sponsors who contribute because they love the animals and the sport. Many major companies and business figures have made, and continue to make, enormous contributions to equestrian sport. One such figure was a recently deceased Swedish industrial leader. Others include well-known Swedish business families and corporate profiles [names removed, author's note]. The pursuit of funding to survive in equestrian sport is therefore entirely understandable. But it is disheartening when well-known riders choose to compete in front of empty stands in the Middle East instead of at home. This is happening more and more often, as oil sheikhs lure them with millions. One solution Falsterbo Horse Show has come up with is to secure a sponsor from the United Arab Emirates. (Ystads Allehanda, February 26, 2025)

Here, the pure money discourse portrays funds from Swedish companies as morally superior, framed as coming from sponsors who “love the animals and the sport,” in contrast to money from countries such as Saudi Arabia or the UAE. Yet it is also entangled with a sense of resignation that dirty money is increasingly preferred. The discourse is further entangled with what Lundström and Hübinette (42) term white melancholia, a cultural condition marked by the feeling that the “good Sweden” is slipping away.

In the media coverage, disappointment over the perceived dissolution of the Swedish self-image can be traced in several statements. In an article from one of the major newspapers in southern Sweden, where the chairman of Falsterbo Horse Show defends the partnership, the journalist writes:

But the chairman of Falsterbo Horse Show is not entirely without a point. He questions why Falsterbo should not be allowed to do what others have been doing for quite some time [names removed, author's note]. Indeed, there is a relatively widespread willingness within Swedish sport to participate in the sportwashing that regimes use to polish their image. Swedish riders regularly compete in the Gulf states without protest, as [name redacted] correctly points out. On the contrary, we cheer when they bring home World Championship and Olympic medals to Sweden. Football executives [names redacted] have applauded Qatar and Saudi Arabia's acquisition of the World Cup. Broadening the perspective, we see Swedish Saab supplying weapons to Saudi Arabia and the UAE. The list is long of blue-and-yellow companies involved in controversial dealings with Gulf states. So why shouldn't a struggling Falsterbo get a piece of the pie? The question is certainly worth asking. At the same time, that kind of whataboutism is rather tiresome. And off the top of my head, I can't think of any Swedish event that has so brazenly tarnished its proud tradition for financial gain. This isn't about a logo on a saddle pad or a named obstacle. It's about the event's very name and identity. It's a new low. And one wonders what's next, now that [names removed, author's note] and the gang have kicked in this door. Gazprom Swedish Open in Båstad? Saudi Aramco Gala at Stockholm Stadium? O-Ringen by Shein? (Helsingborgs Dagblad, March 1, 2025)

In this excerpt, the discourse of pure money operates by mobilizing white melancholia as the writer lists contradictions in Swedish politics and sport. Yet, Falsterbo Horse Show is portrayed as a particularly striking example of something withering away. In doing so, the pure money discourse, entangled with white melancholia, becomes particularly pronounced when it unfolds within Swedish sport. In the country's leading business-oriented newspaper, it is stated:

But one can reasonably argue that sport is something different in Sweden, with roots in popular movements, and that there is often a desire to signal certain values. Whether it's RF [The Swedish Sport Confederation, authors note] or the individual federations, their value statements emphasize democracy, participation, gender equality, diversity, fair play, and inclusivity in a safe environment free from discrimination. Or as the Equestrian Federation writes: “Equestrian sport stands for high safety, ethics, and morality.” Yes, you get the idea, they want to stand for something. But reality tells a different story. From the federation, we've barely heard a whisper about the Al Shira'aa Falsterbo Horse Show. It's like when the Swedish Football Association and 209 other member countries applauded in December to award the World Cup to Saudi Arabia in 2034. The only exception was Norway, which abstained from voting. Some thought it was pointless, but they stood up for their values. (Dagens Industri, March 14, 2025)

In this excerpt, the argument is made about what Swedish sport is expected to uphold: democracy, participation, gender equality, diversity, and fair play, and how these values no longer seem to be honored. It is suggested that Swedish sport, and in this case, particularly the Equestrian Federation, through its silence, relinquish its ideals as well as those of Swedish Sports. In doing so, the pure money discourse mobilizes white melancholia and reinforces the notion that “the good Sweden is gone.”

In this section, I have shown how the discourse of pure money mobilizes nostalgia and national self-idealization. By fetishizing “Swedish money” and invoking white melancholia, the discourse reproduces moral boundaries that position Sweden as inherently virtuous in contrast to morally suspect Others. In this sense, the pure money discourse is deeply entangled with capitalism's identity-producing machinery (7–9).

In the following section, I critically engage with these results, considering previous research and the study's theoretical framework.

## Discussion

In this article, I have examined, from a critical perspective, how the sponsorship agreement between the Falsterbo Horse Show and Al Shira'aa Stables is portrayed in Swedish news media coverage. I show how this portrayal is permeated by three discourses that, in different yet similar ways, operate to frame the sponsorship agreement between Falsterbo Horse Show and Al Shira'aa Stables. These three discourses are: the sold-out soul and sportwashing, dirty money and postcolonial morality, and pure money and Swedish nostalgia. These discourses operate partly by transforming the sponsorship into a geopolitical issue, partly by fetishizing the origin of the sponsor's money, and finally by mobilizing nostalgic sentiments in which Swedish money is imagined as morally pure in contrast to morally suspect funds from the Middle East. These sentiments are accompanied by a sense of loss, a feeling that the “good Sweden” is slipping away. These patterns also resonate with empirical work in sport contexts, showing that commercial arrangements are embedded in the identity-producing machinery and boundary-making practice of capitalism (32–34). Taken together, this empirical work and the present analysis underscore that economic relations in sport actively shape meanings, subjectivities, and legitimacy claims both within sponsorship arrangements and across the wider commercial landscape of sport.

While previous sponsorship research has emphasized strategic, relational, and commercial outcomes, this study shows that sponsorship is also discursively produced through national, postcolonial, and moral imaginaries that shape which sponsors become imaginable or legitimate. This study contributes to sport management and sponsorship research by showing that media portrayals do not merely reflect fans' perceptions of moral appropriateness; they participate in producing these perceptions, in this case by drawing national, postcolonial, and affective boundaries. This boundary-making works by reducing the wider capitalist context of sponsorship to moralized judgments about the origin of money. As a result, portrayals become caught within capitalism's identity-producing machinery (7, 8). In practice, this machinery structures what can be thought, critiqued, or rendered visible, thereby foreclosing alternative forms of critique, for instance, questioning the presupposition that sporting events must rely on sponsorship to be financially viable.

Because sponsorship is embedded within capitalism's identity-producing machinery, these portrayals reinforce the very boundaries and moral distinctions that the system itself generates (7–9). Consequently, certain sponsors' money is framed as dirty and others as pure depending on the geopolitical and cultural context in which the sponsor and sponsored organization are situated, even though all sponsors and sponsored organization are embedded in and benefit from the same capitalist structure. This dynamic is also evident in previous studies, such as Gillooly et al. (2) and Reiche (30), in which some sponsors are perceived as “dirty” by supporters of certain European football clubs but not by others.

The findings also resonate with established sponsorship dynamics. Perceptions that Falsterbo had “sold its soul” mirror typical reactions to name changes (1, 4), and the backlash against Al Shira'aa's Stables presence aligns with a perceived sponsor–property misfit (2, 3, 5, 6, 23), yet the intensity and direction of these reactions are best understood through the national, postcolonial, and affective imaginaries traced above. This perspective helps to show how the responses to the Falsterbo Horse Show—Al Shira'aa Stables sponsorship agreement draw on broader affective and discursive formations that exceed the immediate case.

Among the affective and discursive formations that shape these reactions, white melancholia is particularly helpful. This melancholy is produced by long-standing imaginaries that have understood Sweden and “Swedes” as an egalitarian and progressive nation. This self-image has historically been grounded in Sweden's status as a homogeneous white country in which Swedish whiteness has been associated with moral superiority. Although this self-image is now eroding, a longing for the twentieth century remains (42). The analysis suggests that this longing depends, at least in part, on the continual production of a figure of what Sweden is not, even as Sweden is no longer what it once imagined itself to be. This dynamic becomes visible in the media portrayal of the sponsorship between Falsterbo Horse Show and the Al Shira'aa Stables.

For example, the dirty money discourse operates by emphasizing that Sweden and the UAE hold fundamentally different views on, among other things, gender equality, LGBTQ+ rights, human rights, and animal welfare, where the UAE is framed as traditional while Sweden is framed as progressive. Lundström and Hübinette (42) note, for instance, that the Swedish gender-equality discourse often carries a sense of national exceptionalism, portraying Sweden as a role model in contrast to “others” assumed to be unequal, oppressive, or patriarchal. They also argue that this discourse depends on identifying an “Other”, a body, a culture, or a country that does not share the same values. In a similar way, the pure money discourse operates to suggest that Swedish sponsors would be preferable. This is despite some of Sweden's biggest companies being criticized for ethical shortcomings, environmental negligence, and poor labor conditions, particularly in production in low-wage countries (54, 55). Journalists have also shown how migrant workers in Sweden are exploited under near-slavery conditions, and how refugees from war-torn countries are used as cheap labor without any security (56, 57). White melancholia can also help explain the intensity of the reactions. As Bao (58, p. 200) notes: “If Swedish melancholy is a response to the loss of the imagined attributes that welfare-state nationalism assigns to Sweden and Swedishness—from promises of a caring state, social progressivism, to hegemonic whiteness—then these nostalgic markers are reactivated in every moment of real or perceived crisis.”

Taken together, the analysis shows how the three discourses, the sold-out soul, dirty money, and pure money, operate within capitalism's identity-producing machinery to reproduce moral, national, and postcolonial boundaries. Through processes of fetishization, reduction, and nostalgic self-idealization, the sponsorship agreement becomes a site where anxieties about Sweden's moral integrity and geopolitical standing are mobilized and made visible.

### Implications and future research

The implications of this study are twofold. The first is theoretical: sport management research and sponsorship research would benefit from incorporating more social-scientific and critical perspectives to better understand the cultural and affective contexts in which sponsorship occurs. Such perspectives can, as Chen (31) puts it, create productive opportunities for collective sense-making by making visible the economic and discursive structures that are often taken for granted.

The second implication is practical: those responsible for sponsorship agreements need to develop greater awareness of the societal and discursive contexts in which they are situated, either to avoid controversies like the one between Falsterbo Horse Show and Al Shira’aa Stables, or to better justify and take responsibility for their partnerships.

The study is shaped by its encounters with verbal newspaper text; other media forms would have produced different, not necessarily fuller, lines of analysis. For example, audio material from radio and podcasts discussing sponsorship, as well as visual material from newspapers, could have illuminated additional discursive and affective dimensions of how the sponsorship was represented and interpreted in the media. Future research could build on this by examining how capitalism's identity-producing machinery operates across various sporting contexts and media environments. This could offer deeper insights into how economic relations in sport not only organize resources, but also produce affects, identities, and imaginaries of nation, morality, and belonging. Future studies could also explore how these discourses are understood and negotiated by differently situated individuals, for example, along lines of age, gender, ethnicity, sexuality, and socio-economic background, to shed light on how moral and national imaginaries are taken up in practice.

## Data Availability

The original contributions presented in the study are included in the article/Supplementary Material, further inquiries can be directed to the corresponding author.
